# The Institutional Problem of Mental Defectives

**Published:** 1914-11-21

**Authors:** 


### THE INSTITUTIONAL PROBLEM OF MENTAL DEFECTIVES.

LAST week we drew attention to a suggestion of the Mental Hospitals Committee of the London County Council that accommodation for the various classes of mental defectives should be provided by the Council in special institutions of its own. As a beginning it was decided to purchase the Mission Schools for Hebrew Children at Streatham Common and adapt them for the reception of seventy feeble-minded girls and young women. This is the first step in a reform full of promise for the amelioration of the position of a most unfortunate section of the community, and one which will probably lead to other much needed improvements. The problem of the mental defectives in London is complicated by the number of authorities having control of them. Provision for them in public institutions is made by the London County Council, the Metropolitan Asylums Board, and the various Boards of Guardians. It is one of the defects of the Mental Deficiency Act, 1913, that this multiplication of controlling authorities is perpetuated. There is no system of distribution of the various classes of mental defectives amongst these authorities, and it is to a great extent a matter of chance whether a given patient falls to the care of one or other of them.

At present the vast majority of the poorer cases are admitted in the first place to the Poor-Law infirmary or workhouse. Here the worst cases are certified and most of them transferred to other institutions. Those showing violent or suicidal tendencies are as a rule transferred to the asylums of the London County Council; children and harmless adults are sent to the institutions of the Metropolitan Asylums Board; quiet patients whose mental defect arises from senile decay or other physical infirmities are usually treated in the infirmary or workhouse; and, lastly, the best types of adult imbeciles and feeble-minded are often kept as useful workers. It is difficult to determine from the statistics published by the Lunacy Commissioners how many "*certified* lunatics" are thus retained in workhouses and infirmaries, but a careful examination and collation of the various tables shows that the number is comparatively small. A return obtained by the Local Government Board on July 20, 1912, shows, however, a large number of *uncertified* mental defectives in the various institutions of the Metropolitan Boards of Guardians. This return is based upon the definitions of mental defect contained in the Mental Deficiency Bill of 1912. In the Act of 1913 these definitions were modified, and one large class, the mentally infirm—that is, those whose mental defect is due to age or the decay of their faculties—was entirely excluded. However, the return gives a fairly accurate idea of the numbers of the four classes of mental defectives coming within the scope of the Mental Deficiency Act under the care of Metropolitan Boards of Guardians at the date mentioned. There were 38 idiots, 220 imbeciles, 1,761 feeble-minded persons, and 92 moral imbeciles. A small proportion of the total were in the receipt of outdoor relief, in institutions not under the control of any Poor-Law authority, or in the asylums of the Metropolitan Asylums Board, but most of them were in the workhouses and infirmaries.

It is obvious that the great majority of these cases ought to have been in specialised institutions. Every Poor-Law medical officer knows the need there is for them, and would gladly avail himself of the opportunity of transferring his mental defectives to them if they were in existence. The necessary accommodation could be obtained without any very great expense by taking over some of the small workhouses in the neighbourhood of London. There are several which, with a little adaptation, would make excellent colonies, their present inmates being accommodated in the workhouses of adjoining parishes.

We believe, however, that the institutional treatment of lunacy and mental deficiency in London will never be efficient and economical until the London County Council is made the sole authority for its provision, and the mental hospitals of the Metropolitan Asylums Board are transferred to it.

